# State of the art in AIT: The patients’ perspective 

**DOI:** 10.5414/ALX02273E

**Published:** 2022-04-08

**Authors:** Sabine Jossé, Kymble Spriggs

**Affiliations:** 1 MeinAllergiePortal, Kronberg, Germany, and; 2 Department of Medicine, The University of Melbourne, Melbourne, Australia

**Keywords:** AIT, patients’ view, allergy, patient-centered care

## Abstract

Background: On the occasion of the 110^th^ anniversary of allergen-specific immunotherapy (AIT), the question arises of “how do patients feel about AIT?”. Materials and methods: Informed by questions and feedback provided to MeinAllergiePortal, an online survey with a target of 130 responses was devised and offered for completion by readers. All visitors of MeinAllergiePortal categories addressing inhalant allergies were invited to participate in the survey. Participants were grouped and analyzed by their AIT completion status. The survey was ended once target was met. Results: 121 of 132 participants were familiar with AIT. 1) A majority of patients who completed AIT would choose the therapy again; 2) Physicians do not seem to discuss AIT with all patients with significant symptoms; 3) Adverse reactions appear to be a major reason why patients terminate AIT prematurely; 4) Lack of time, or early response, as often supposed, does not seem to be a major factor leading to discontinuation of AIT. Conclusion: Patients’ experience and understanding of symptoms (both related to allergic disease, or expected AIT-related adverse events) appear to be key factors related to AIT engagement and adherence. Given the importance of adherence for AIT efficacy, improved education and support strategies may assist patients achieve their treatment goals.

## Introduction 

In 2021, allergen-specific immunotherapy (AIT) celebrated its 110^th^ anniversary. It was Leonard Noon, who first described this new therapeutic concept for the treatment of grass pollen allergy in “The Lancet” in 1911 [1]. Since then, AIT has been intensively researched and administered. Allergologists and clinical trial data agree, that AIT can be an effective therapy for a variety of IgE-mediated allergic diseases. In addition to this, AIT is considered as “the only causal therapy” [2] for certain allergies. 

Today, the application of AIT is not restricted to the treatment of grass pollen allergy. Many other allergic diseases can be treated with AIT, and there is wide ongoing research on further applications. 

According to the most recent AIT guideline [3], indications for AIT are inhalant allergies against:

- grass pollen
- tree pollen
- house dust mites
- animal allergens

Furthermore, AIT is indicated for patients with systemic reactions to bee venom or wasp venom, and since 2018, “NVL (Nationale Versorgungsleitlinie) Asthma” is recommending AIT for the treatment of asthma [4, 5]. And only recently the first oral AIT for the treatment of peanut allergy has been approved by the FDA [6]. 

While AIT is still commonly administered via “classical” subcutaneous injections, the variety of administration options has increased substantially. Today, AIT can be administered as a fast track version in form of an ultra-rush immunotherapy. For certain indications, AIT can be administered as an oral or sublingual immunotherapy (SLIT) via tablets or drops. Subsequently, patients now have the option to choose a therapeutic pathway that suits their personal everyday lives. These options are associated with increasing use of AIT [7]. 

However, in spite of this, it is estimated only 10% of allergic patients are treated to guidelines [8]. 

A survey conducted in 2016 with 15,000 participants indicated that only 31.7% of the respondents eligible for AIT actually received the therapy [9]. Another German population survey of 1,000 individuals representative of the underlying population yielded similar results (29%) [10]. 

These data suggests that a substantial number of allergy patients are not receiving otherwise appropriate AIT. There might be many reasons for this, but the patient likely plays an important role, as the authors of the latest AIT guideline emphasize, stating that AIT “is heavily dependent on patient compliance” [3]. 

The question is: What is the patients’ perspective on AIT? 

## Materials and methods 

MeinAllergiePortal is an online portal, mainly for patients, focusing on providing medically correct information on allergic diseases in German language. It was founded in 2013 and is independent of pharmaceutical or media groups. In 2020, the portal was visited by 2.4 million readers – many of which give their opinion on various aspects of allergic diseases. This feedback informed the editorial team as to important aspects of the patients’ perception and thereby survey design. 

The survey was conducted June 30 to July 20, 2021 on MeinAllergiePortal, asking participants for their opinions on AIT. All visitors of MeinAllergiePortal categories addressing inhalant allergies were invited to participate in the survey. During the above time period the relevant pages had ~ 14,000 visitors. We addressed inhalant allergies in general in our survey, not only pollen allergies, and focused on the patients’ past experiences. There might be seasonal fluctuations, but we did not consider them relevant. Our goal was to get rapid feedback of at least 130 respondents, and we stopped the survey when that goal was met. In patients with an understanding of AIT, survey statements were presented for patients to agree or disagree. These statements were dynamic and adjusted depending on participants treatment status, i.e.:

- treated with AIT and completed the course
- treated with AIT but ceased treatment early
- not yet been treated with AIT

## Results 

121 of 132 respondents stated that they were familiar with AIT as a therapy for allergic diseases. 

**Figure 1:**
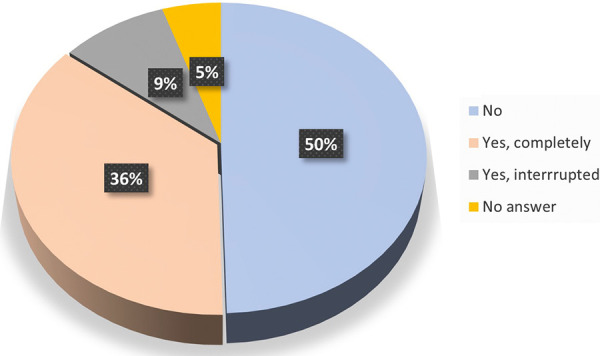
Survey participants familiar with AIT. Have you personally undergone an AIT?

Figure 1 represents those who were familiar with AIT – 45% stated that they personally had already undergone AIT; 50% had not; and 5% made no statement. Among the respondents who had already undergone AIT, 80% had completed the therapy. 

### The patients’ perspective: In those whose AIT was carried out to completion (Figure 2) 

73% of those completing AIT would “choose AIT as an allergy treatment any time again”. But: More than 1 in 4 AIT users would not. 

63.4% of those completing AIT treatment stated that they considered AIT as “an appropriate therapy”. 

Only 33.3% of those who had finished AIT treatment claimed that they were “completely or almost completely cured by AIT”. 

Significant adverse reactions did not seem to be a problem. Only 19.5% claimed that they had experienced those. 80,5% did not report any side effects. 

Information provided by the patients’ physicians was largely assessed as sufficient. The majority of those surveyed, 87.5%, declared that they had been “very well informed and looked after” by their physician. 

### The patients’ perspective: In those whose AIT was started but ceased early (Figure 3) 

More than a quarter of those ceasing treatment early (28.6%) agreed that they were not looked after well by the treating physician, leading to premature termination. 

Early success in treatment was never given as a reason for early termination. 

Similarly, no participants indicated time required for participation as a reason for early termination. 

By far the most common reason for canceling AIT therapy before it was finished was “adverse reactions”. As many as 90.9% of those surveyed stated that this was the reason. 

The second most “agreed with” reason for interrupting the treatment was that “AIT was not successful”. 42.9% of the surveyed participants agreed to this statement. 

### The patients’ perspective: Obstacles to AIT? (Figure 4) 

A rather interesting result of the MeinAllergiePortal survey was the most common answer to the question why those who knew about AIT had not been treated with it so far. 

The main reason for 77.1% of those surveyed was that their physician had not offered AIT as a therapy. 

The second most common answer, given by 40.4% of the surveyed, was that patients perceived their allergic symptoms as “too minor for AIT to be necessary”. Whether this is the patients’ assessment or the physicians’ advice cannot be deduced. 

For one third of the participants (33.3%) “fear of side effects” was a reason why they have not started AIT. 

28.2% thought a lack of time would prevent them from starting AIT. 23.8% are “not convinced of AIT’s prospects of success”. 

In contrast to what one might have expected, the COVID-19 pandemic appears not to have significantly influenced the patient view on AIT. We asked a single question “Has Corona changed your view on AIT?” [yes/no] after the above set of questions. 96% answered “No” – i.e., that their opinion on AIT had not been changed by coronavirus. 

## Discussion 

Although the underlying details of these patients’ treatment courses are not available, the results of this survey provide quite a striking insight into their perspectives of AIT and potentially important factors affecting treatment commencement, adherence, and thereby success. Although the underlying details of these patients’ treatment courses are not available, the pseudo-stratification (AIT completion, partial completion, and those who have not yet commenced) provides quite a striking insight into their perspectives of AIT and potentially important factors affecting treatment commencement, adherence, and thereby success. 

In those fully completing therapy, perhaps unsurprisingly, the majority (73%) agreed they would choose AIT again as a treatment. Despite this apparent enthusiasm, only one third (33%) agreed that AIT “cured or largely cured” their allergy. This apparent disparity may parallel the challenges in defining efficacy in clinical trials – and the fact that patient-reported “quality of life” outcomes rather than measurement of allergy symptoms in isolation are increasingly being considered as meaningful endpoints. On the other hand, this could be due to potential waning of effects over time (which may affect patients’ assessment of “cure”), as we have no information of how long ago patients participating in the survey received their AIT. 

What is important may differ between individuals and may need to be identified in collaboration with the physician for a specific patient. 

In those completing the full therapy, by far the majority (87.5%), felt well cared for by their physician – potentially a supporting factor for completion. 

Of those who ceased their AIT treatments early, about twice the proportion of patients did not feel well looked after (28.6%) compared to continuing patients. 

However, the vast majority (90.9%) of those terminating early agreed that adverse reactions caused them to interrupt therapy. Even without knowing the specific details of the symptoms involved, this appears a large number compared to expected rates [11]. Whether those adverse reactions were extremely severe, or whether the mere fact that there were adverse reactions at all, causing those patients to quit AIT is unknown. But it does raise questions as to how thoroughly those patients were informed regarding the expected nature of adverse reactions in AIT and their significance, and how proactively these were managed. Potentially improved patient education may meaningfully affect expectations and the patient experience and therefore early cessation/outcomes. 

Amongst those who ceased early, a larger proportion (42.9%) agreed that AIT was not successful for them. Although this is not clear, it implies that the simple majority (57.1%) may have found it successful but ceased anyway (perhaps due to adverse reactions as above). 

We also have no data on expectations on the patients’ side, particularly if they were informed about the degree of effectiveness to be expected by AIT. Did they know about when exactly the treatment usually begins to work? Was AIT supposed to control symptoms completely or rather mitigate them and reduce medication dosages? All of these answers would give significant context to these statements – and may be profoundly affected by appropriate education. Perhaps surprisingly, early allergy symptom improvement (leading to cessation), or the time demands required by therapy, were selected by none (0%) of the participants who ceased early. Anecdotally, these are thought to be factors affecting ongoing adherence, so their complete absence within this survey appears significant. 

Of those who have not yet started AIT so far, the most common agreed statement (77.1%) was that their physician had not suggested AIT as a therapy. One possible explanation might be that treating physicians do not carry out this therapy in their own practice and are hesitant to refer their patients to specialized centers. This theory is supported by general feedback MeinAllergiePortal receives from its readers on a regular basis: Patients often approach MeinAllergiePortal after having read about certain therapies or diagnostic tools on the website, asking which physician to turn to when their own physician cannot provide help. Physician as a “gate-keeper” appears to be a significant barrier to access for AIT, as of the patients not on therapy a large majority have not even had it suggested as an option. 

Reasonable patient concerns around adverse reactions/risks (33%), time required (28%), success rates (24%) were measured, all of which may be addressable by patient education. 

The second most common answer, given by 40.4% of the surveyed, was that patients agreed their allergic symptoms as “too minor for AIT to be necessary”. Whether this is the patients’ assessment or the physicians’ advice cannot be deduced. 

Pollen allergy sufferers who approach MeinAllergiePortal for advice often report that their symptoms vary greatly from season to season. They may not see a need for AIT as long as they do not suffer from allergy symptoms continuously. When asked about the risk that pollen allergies could develop into asthma or that multiple allergies may develop, it often turns out that patients are not aware of this. There appears to be room for increased patient awareness. However, the converse situation for the majority (i.e., almost 60% have symptoms that are not considered minor) again suggests perceived barriers as above are preventing significantly symptomatic patients from receiving treatment. 

The limitations of an optional website-based survey is by its very design likely an incomplete sample of the wider patient population affected by AIT. A selection bias may have also been introduced by virtue of the survey being only presented to those participants searching for online information in that particular month. 

Recall and observational biases are very likely present, although their direction and magnitude are difficult to assess in the small sample set. Other surveys of AIT patients have previously documented specific lack of recall on key AIT points [12]. 

However, despite these biases, the survey of participants of MeinAllergiePortal is unique as it gleans perspectives from an AIT-engaged population. These data may inform future enquiries in this area. 

## Conclusion 

Patients’ experiences of symptoms (due to both allergic disease and expected therapy adverse events) appear to feature strongly in the patients’ perspective of AIT. Perceptions, expectations and meaning attributed to these symptoms may to be associated with initial engagement and adherence to therapy. 

Due to the medium-term nature of AIT, engagement and participation/adherence is key to effectiveness. 

These data may suggest that education and guided support around experienced symptoms could be important to maximize adherence – especially as symptoms are expected to change significantly over the duration of AIT and the responses may vary significantly between individuals. Thus, improved education and support strategies may assist patients achieve their treatment goals. 

Further research into such strategies for effective patient education is warranted to support effective use of AIT. 

## Funding 

No external funding was received. 

## Conflict of interest 

KS is a practicing allergologist and clinical immunologist & has previously received honoraria and grants from Stallergenes-Greer, Seqirus. 

SJ is co-founder of Mein-Allergie-Portal an online health resource for allergic diseases & patients. 

**Figure 4:**
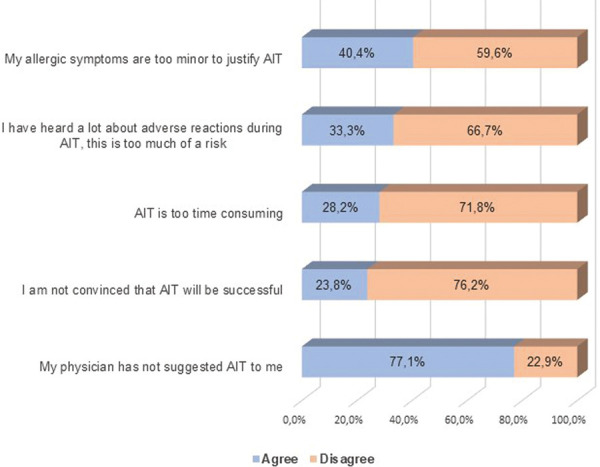
Survey participants who are familiar with AIT but have not started a therapy yet. What are the reasons for not starting AIT? Please indicate whether you agree or disagree with the following statements.

**Figure 3:**
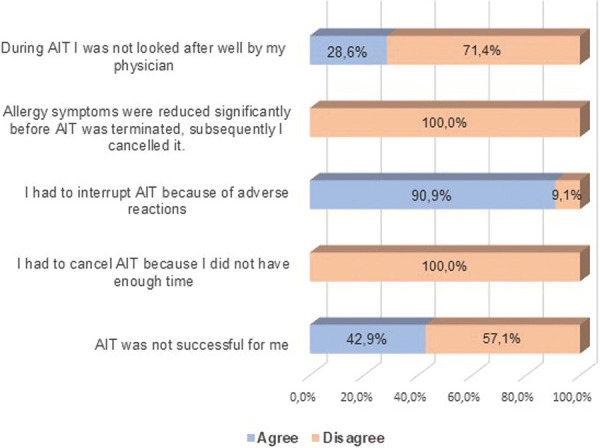
Survey participants that canceled AIT Why was AIT terminated prematurely? Please indicate whether you agree or disagree with the following statements.

**Figure 2:**
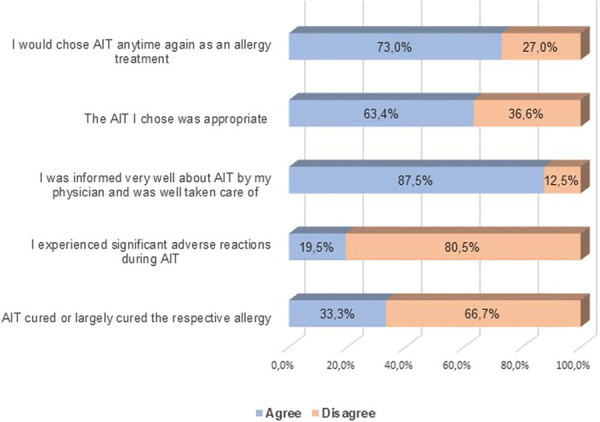
Survey participants who completed AIT: What is your view on AIT? Please indicate whether you agree or disagree with the following statements.
